# Understanding Delays and Diagnostic Shifts in Primary Headaches: Evidence from Japanese Health Insurance Claims

**DOI:** 10.7759/cureus.85005

**Published:** 2025-05-28

**Authors:** Yuki Tatsuno, Masahito Katsuki, Yumi Kawata, Takao Takeshima

**Affiliations:** 1 Medical Affairs, Hedgehog MedTech, Inc, Tokyo, JPN; 2 Insight Science Foundation Ireland Research Centre for Data Analytics, School of Human and Health Performance, Dublin City University, Dublin, IRL; 3 Physical Education and Health Center, Nagaoka University of Technology, Niigata, JPN; 4 Neurology, Headache Center, Department of Neurology, Tominaga Hospital, Osaka, JPN

**Keywords:** diagnostic delay, migraine disorder, primary headache disorder, underdiagnosis, misdiagnosis

## Abstract

Introduction

The early and accurate diagnosis of primary headaches is essential for improving patients’ quality of life and preventing the overuse of acute medications. Lack of a proper diagnosis not only reduces the patients’ quality of life but also may result in patients having to return to other medical facilities or undergo duplicate tests, which has a negative impact on the health care system, including increasing medical costs. In Japan, although primary headaches are prevalent, studies investigating the misdiagnosis and underdiagnosis of headaches are limited. This study aimed to explore current trends in primary headache diagnosis and investigate how these diagnoses changed during the five years following the initial diagnosis.

Methods

We used an anonymized, nationwide health insurance claim database from Japan, which contained data from July 2010 through November 2024. We included the outpatient claim data of adults aged 18 years or older. Using the International Statistical Classification of Diseases and Related Health Problems, 10th Revision (ICD-10), we classified headache diagnoses into five categories: migraine and medication-overuse headaches (MOHs), tension-type headaches (TTHs), trigeminal autonomic cephalalgia (TACs), other primary headaches, and other headaches including secondary headaches. Cases with no specific headache diagnosis based on the ICD-10 were classified as unconfirmed. We assessed diagnostic consistency between the initial diagnosis and 3, 6, 12, 36, and 60 months later.

Results

We analyzed data from 336,596 patients. The mean (SD) age was 40.2 (12.3) years. Overall, 91,830 patients (27.3%) were men and 244,766 (72.7%) were women. Regarding the initial diagnoses, 95,890 (28.5%) were unconfirmed diagnoses, 181,651 (54.0%) were migraine or MOH, 56,296 (16.7%) were TTH, 853 (0.3%) were TACs, 1,663 (0.5%) were other primary headaches, and 243 (0.1%) were other headaches. Of the 86,164 patients (25.6%) who had a definitive headache diagnosis three to five years after their initial visit, 51,360 (59.6%) received the same diagnosis at their initial visit and subsequent visit. The most common headache diagnosis after three to five years was migraine or MOH (n=67,939; 78.8%), followed by TTH (n=17,371; 20.2%), other primary headaches (n=488; 0.6%), TACs (n=307; 0.4%), and other headaches (n=59; 0.07%). The most frequent diagnostic change was from a TTH diagnosis to a migraine or MOH diagnosis (n=2,522; 2.9%).

Conclusions

Over one in four patients received an unconfirmed diagnosis during their initial visit and more than 40% of patients were given different diagnoses during a later follow-up. These findings indicate the challenges and delays associated with reaching a definitive diagnosis for primary headaches.

## Introduction

Primary headaches, defined as headaches not related to an underlying medical condition, are classified into four categories based on the International Classification of Headache Disorders (ICHD-3): migraine, tension-type headache (TTH), trigeminal autonomic cephalalgias (TACs), and other primary headache disorders [1]. Primary headache disorders are common, with some studies estimating that globally 40% of adults experience headache disorders [2]. A previous study reported that 14% experienced migraines, 26% experienced TTH, and 4.6% experienced chronic daily headaches [3]. Despite their widespread impact, particularly among young and middle-aged females, the public health importance of primary headaches has been underrecognized [4].

The timely and accurate diagnosis of headaches is essential for the delivery of appropriate acute and prophylactic treatment, and for reducing the intensity, frequency, and overall burden associated with headaches. Inadequate treatment due to incorrect or delayed diagnoses can lead to unmanaged pain and medication overuse headaches (MOHs). One primary headache that is greatly affected by the lack of a correct diagnosis is migraine. Migraines are particularly burdensome due to their negative socioeconomic impacts [5] and the risk of progression to chronic migraines, which are particularly difficult to manage, often requiring more intensive care [6]. To assist physicians in making correct diagnoses, guidelines such as the ICHD-3 and multiple diagnostic tools, including screening questionnaires, should be used [1,7,8]. However, several studies have highlighted persistent challenges and delays regarding headache diagnoses, particularly in primary care settings [7,9-11]. Lack of comprehensive training in headache diagnosis and treatment may be a contributing factor [11].

In addition to the direct impact on patients, previous studies have pointed to the impact on healthcare costs. Patients frequently consulted numerous doctors but despite the repeated visits, many patients remained undiagnosed. The following physician often performs duplicates or repeats tests performed by the previous physician. Patients with chronic headaches, particularly migraines, often experience increased healthcare utilization and associated costs due to misdiagnosis, repeated hospital visits, and duplicate testing [12].

In Japan, primary headaches are also prevalent and burdensome [13,14]. Previous studies in Japan also indicated the difficulties in diagnosing primary headaches correctly because there are no objective tests and biomarkers and diagnoses should be made based on subjective symptoms, which can be changed over time. For example, only 11.6% of migraine patients were correctly diagnosed as such, while the mean time to a correct diagnosis for cluster headaches (a type of TACs) was 7.3 years [15,16]. Despite this evidence, few studies have investigated the diagnosis of primary headaches at the national level. Understanding diagnostic patterns may help in the development of interventions aimed at improving diagnostic speed and accuracy, while reducing missed diagnoses. Therefore, this study aimed to clarify how frequently and in what ways headache diagnoses change over time following a patient’s initial visit, using nation-wide insurance claim data to uncover the extent of diagnostic delays and inconsistencies in primary headache care.

## Materials and methods

Data

In Japan, all residents are required to be covered by health insurance provided by either employer- or community-based insurers [17]. The REZULT database, offered by Japan System Techniques Co., Ltd. Tokyo, Japan [18], is a health insurance claim database of employee-based insurers, which covers over nine million patients. The database includes inpatient, outpatient, and pharmacy claims from July 2010 through November 2024, which was used as the study period. Each patient is assigned a unique anonymized identification number, enabling longitudinal observations of headache diagnoses even when patients visit different medical institutions. In Japan, insurance claims are typically submitted using diagnoses based on the International Statistical Classification of Diseases and Related Health Problems, 10th Revision (ICD-10). Accordingly, this study extracted data based on the ICD-10 codes relevant to headaches, specifically codes of R51, G43, and G44, even though ICHD-3 classifications do not align with the ICD-10 codes [19].

Population

We conducted a retrospective analysis of patients aged 18 years or older who had headaches diagnosed at least once during the study period. Diagnoses were defined using the ICD-10 codes. In the Japanese health insurance claim system, physicians can label a diagnosis as “suspected”; diagnoses without this label are considered definitive. We included both suspected diagnoses and definitive diagnoses to capture a broad range of headache diagnoses during the initial visits; however, we excluded patients who have never received any definitive diagnoses or those who only received unconfirmed headache diagnoses at the subsequent visits. To assess how headache diagnoses changed over time, we excluded patients who had visited medical institutions for headaches only once. We excluded inpatient and dental claims.

Headache diagnosis categories

We classified headache diagnoses into five categories based on ICD-10 codes. The migraine and MOH codes were G43, G430, G431, G433 G444, and G439, the TTH code was G442, the TACs code was G440, the other primary headaches code was G448, and the other headaches codes (including secondary headaches) were G441 and G443 [1]. We defined a definitive headache diagnosis as one that fell into one of these five categories and had no “suspected” label. We also defined unconfirmed diagnoses as R51 and G44, with the third digit not specified.

Outcome variables

The primary outcome was the consistency of diagnoses between initial and subsequent visits. Assuming that later definitive diagnoses represented the most accurate diagnoses, we calculated the accuracy rate based on the number of patients whose diagnoses remained consistent between their initial and subsequent visits divided by the total number of patients. Subsequent visits were categorized into different periods: one to three months, four to six months, seven to 12 months, 13-36 months, and 37-60 months according to the previous study assessing the time to reach the correct diagnosis. The most recent and definitive diagnosis was used when more than one diagnosis was registered during the period. When different types of headaches were registered simultaneously, diagnoses were prioritized in the order of migraine and MOH, TTH, TACs, other primary headaches, and other headaches. The secondary outcomes were the accuracy rate, sensitivity, specificity, and positive predictive value (PPV) of each headache diagnosis category. These secondary outcomes sought to clarify whether changes in diagnoses differed depending on the headache diagnosis category, assuming that later definitive diagnoses were correct.

Statistical analysis

First, we analyzed the characteristics of patients and medical institutions during the initial visits. Institutional characteristics included location (Eastern Japan: Hokkaido, Tohoku, and Kanto, Central Japan: Chubu and Kansai, and Western Japan: Chugoku, Shikoku, and Kyushu/Okinawa), department or specialties listed, and number of the beds. Detailed definitions of the department categorizations are described in Table 1.

**Table 1:** Categorization of department specialities at medical institutions Medical institutions were categorized into one of three groups based on their declared department specialties: (1) internal medicine, (2) non-internal medicine, and (3) unknown. In Japan, physicians can establish clinics regardless of their specialty and any board certification status. The database used only included the primary department declared by each institution. No data were available regarding the specialties of the physicians who made the headache diagnoses.

Category	Department speciality
Internal medicine	Allergic diseases; Cardiovascular disease; Diabetes; Dialysis medicine; Endocrinology and metabolism; Gastroenterology; General internal medicine; Nephrology; Neurology; Pediatrics; Pulmonary disease; Rheumatology
Non-internal medicine	Anaesthesiology; Breast surgery; Cardiovascular surgery; Chinese medicine; Colorectal disease; Dentistry; Dermatology; Emergency medicine; Gastroenterological surgery; Gastrointestinal endoscopy; General surgery; Neurosurgery; Obstetrics and gynecology; Ophthalmology; Orthopedics; Otorhinolaryngology; Pain medicine; Pediatric psychiatry; Pediatric surgery; Physiatry (rehabilitation); Plastic surgery; Psychiatry; Psychosomatic medicine; Radiology; Urology; Others
Unknown	Missing

Second, we analyzed the diagnoses given during initial visits. Third, we calculated the overall accuracy rate of headache diagnoses between the initial and subsequent visits among patients who had definitive diagnoses during the period. Fourth, we calculated the accuracy rate, sensitivity, specificity, and PPV of each headache diagnosis category. Stata version 18 (Stata Corp., College Station, TX, USA) was used for all analyses.

Subgroup analyses

To explore how patients were diagnosed, we analyzed changes in headache diagnoses among patients whose initial diagnoses were unconfirmed. We analyzed when patients visited a medical institution for the second time, and the length of time until they had a definitive headache diagnosis of migraine and MOH, TTH, TACs, other primary headaches, or other headaches.

Ethics

The requirement for written informed consent was waived due to the retrospective nature of this study and the use of anonymized data. All procedures were conducted in accordance with the ethical principles outlined in the Declaration of Helsinki and adhered to the Strengthening the Reporting of Observational Studies in Epidemiology (STROBE) guidelines.

The REZULT database, developed by Japan System Techniques Co., Ltd., was used. This database comprises anonymized health insurance claims data provided by insurers. As the data are anonymized and do not contain personally identifiable information, individual patient consent was not required for its secondary use, in accordance with the Act on the Protection of Personal Information in Japan. Furthermore, because the study used anonymized commercial data, it is not subject to institutional ethical review under the Japanese Ethical Guidelines for Medical and Health Research Involving Human Subjects.

## Results

Study population characteristics 

We analyzed 336,596 patients. The mean age was 40.2 years (SD 12.3), and 91,830 patients (27.3%) were men, while 244,766 (72.7%) were women (Table 2).

**Table 2:** Patient and medical institution characteristics at the time of initial visit ^a^The numbers are no. (%), except for age. ^b^We classified headache diagnoses into five categories based on the ICD-10 codes: unconfirmed (R51, G44), migraine and medication-overuse headache (MOH); (G43, G430, G431, G432, G433, G438, G444, G439), tension-type headache (TTH; G442), trigeminal autonomic cephalalgias (TACs; G440), other primary headaches (G448), and other headaches including secondary headaches (G441, G443). ^c^Clinic/hospital characteristics are based on the characteristics of the medical institutions that recorded the headache diagnoses initially after patients were included in the database.

		N (%)
Patient characteristics		
No. of patients		336,596
Age , mean (SD), years		40.2 (12.3)
Sex at birth	Female	244,766 (72.7)
	Male	91,830 (27.3)
Initial diagnosis^b^	Unconfirmed	95,890 (28.5)
	Migraine or MOH	181,651 (54.0)
	TTH	56,296 (16.7)
	TACs	853 (0.3)
	Other primary headaches	1,663 (0.5)
	Other headaches	243 (0.1)
Region^c^	Hokkaido/Tohoku	31,416 (9.3)
	Kanto	121,198 (36.0)
	Chubu	50,217 (14.9)
	Kansai	56,392 (16.8)
	Chugoku/Shikoku	22,871 (6.8)
	Kyusyu/Okinawa	43,783 (13.0)
	Unknown	10,719 (3.2)
Department^c^	Internal Medicine	196,178 (58.3)
	Non-internal medicine	129,696 (38.5)
	Unknown	10,722 (3.2)
No. of beds^c^	0	221,174 (65.7)
	1-19	22,036 (6.6)
	20-299	41,373 (12.3)
	≥300	41,294 (12.3)
	Unknown	10,719 (3.2)

During the initial visit, 58.3% of the patients presented to the internal medicine department and 38.5% presented to a non-internal medicine department. The medical institutions patients presented to for the initial visit were in Eastern (45.3%), Central (31.7%) and Western Japan (19.8%). Most patients visited clinics without inpatient beds (65.7%).

Initial headache diagnoses

During their initial visit, 95,890 patients (28.5%) received an unconfirmed headache diagnosis. Migraine or MOH was the most common initial diagnosis (n=181,651, 54.0%), followed by TTH (n=56,296, 16.7%), other primary headaches (n=1,663, 0.5%), TACs (n=853, 0.3%), and other headaches (n=243, 0.1%), as shown in Table 2.

Headache diagnoses after three to five years

After excluding patients whose diagnoses were not re-recorded three to five years after their initial visit, 86,164 patients (26.0%) had a definitive headache diagnosis. As shown in Table 3, the most common headache diagnosis after three to five years was migraine or MOH (n=67,939, 78.8%), followed by TTH (n=17,371, 20.2%), other primary headaches (n=488, 0.6%), TACs (n=307, 0.4%), and other headaches (n=59, 0.07%). The diagnostic accuracy rate was 59.6%, meaning that 51,360 patients received the same diagnosis at their initial and subsequent visits. 

**Table 3:** Consistency of initial headache diagnoses and diagnoses after 3–5 years ^a^Subsequent diagnoses were defined as the latest diagnoses recorded between 37 and 60 months after initial diagnosis. ^b^We classified headache diagnoses into five categories based on the ICD-10 codes: unconfirmed (R51, G44), migraine and medication-overuse headache (MOH); (G43, G430, G431, G432, G433, G438, G444, G439), tension-type headache (TTH; G442), trigeminal autonomic cephalalgias (TACs; G440), other primary headaches (G448), other headaches including secondary headaches (G441, G443). Initial diagnoses included suspected diagnoses; however, subsequent diagnoses excluded suspected diagnoses.

	Diagnosis after 3–5 years^a^						Indicators of each headache category			
Initial diagnosis	Migraine or MOH^b^	TTH^b^	TACs^b^	Other primary headaches^b^	Other headaches^b^	Total	Accuracy rate (%)	Sensitivity (%)	Specificity (%)	Positive predictive value (%)
Unconfirmed^b^	22,277	7,289	85	234	41	29,926	-	-	-	-
Migraine or MOH^b^	42,960	1,883	105	67	7	45,022	68.6	63.2	88.7	95.4
TTH^b^	2,522	8,139	17	30	5	10,713	86.3	46.9	96.3	76.0
TACs^b^	96	15	99	1	0	211	99.6	32.2	99.9	46.9
Other primary headaches^b^	81	40	1	156	0	278	99.5	32.0	99.9	56.1
Other headaches^b^	3	5	0	0	6	14	99.9	10.2	99.99	42.9
Total	67,939	17,371	307	488	59	86,164	59.6	-	-	-

The accuracy rate, sensitivity, specificity, and PPV of the migraine and MOH category were 68.6%, 63.2%, 88.7%, and 85.4%, respectively. Although the accuracy rate and specificity of migraine and MOH were the lowest of the five headache categories, the sensitivity and PPV of migraine and MOH were the highest. Of the patients whose initial diagnosis was unconfirmed, the most common diagnosis at their subsequent visit was migraine and MOH, followed by TTH, other primary headaches, TACs, and other headaches. Of the patients who received a specific diagnosis at their initial visit, the most common change in diagnosis during the subsequent visit was from TTH to migraine or MOH (n=2,522 patients, 2.9%). 

Headache diagnoses after one to three months, four to six months, seven to 12 months, and 13-36 months 

The diagnoses during the follow-up periods (one to three months, four to six months, seven to 12 months, and 13-36 months after the initial visit) are described in Tables 4-7.

**Table 4:** Consistency of initial headache diagnoses and diagnoses after 1–3 months ^a^Subsequent diagnoses were defined as the latest diagnosis recorded between one and three months after the initial diagnosis. ^b^We classified headache diagnoses into five categories based on the ICD-10 codes: unconfirmed (R51, G44), migraine and medication-overuse headache (MOH); (G43, G430, G431, G432, G433, G438, G444, G439), tension-type headache (TTH; G442), trigeminal autonomic cephalalgias (TACs; G440), other primary headaches (G448), other headaches including secondary headaches (G441, G443). Initial diagnoses included suspected diagnoses; however, subsequent diagnoses excluded suspected diagnoses.

	Diagnosis after 1–3 months^a^						Indicators of each headache category			
Initial diagnosis	Migraine or MOH^a^	TTH^a^	TACs^a^	Other primary headaches^a ^	Other headaches^a^	Total	Accuracy rate (%)	Sensitivity (%)	Specificity (%)	Positive predictive value (%)
Unconfirmed^b^	9,049	3,218	46	110	15	12,438	-	-	-	-
Migraine or MOH^b^	114,928	860	67	39	5	115,899	93.0	91.8	97.3	99.2
TTH^b^	1,144	29,861	2	11	0	31,018	96.7	87.9	99.1	96.3
TACs^b^	73	11	436	1	0	521	99.9	79.1	99.9	83.7
Other primary headaches^b^	52	17	0	787	0	856	99.9	82.9	99.96	91.9
Other headaches^b^	3	4	0	1	116	124	99.98	85.3	99.99	93.5
Total	125,249	33,971	551	949	136	160,856	90.8	-	-	-

**Table 5:** Consistency of initial headache diagnoses and diagnoses after 4–6 months ^a^Subsequent diagnoses were defined as the latest diagnosis recorded between four and six months after the initial diagnosis. ^b^We classified headache diagnoses into five categories based on the ICD-10 codes: unconfirmed (R51, G44), migraine and medication-overuse headache (MOH); (G43, G430, G431, G432, G433, G438, G444, G439), tension-type headache (TTH; G442), trigeminal autonomic cephalalgias (TACs; G440), other primary headaches (G448), other headaches including secondary headaches (G441, G443). Initial diagnoses included suspected diagnoses; however, subsequent diagnoses excluded suspected diagnoses.

	Diagnosis after 4–6 months^a^						Indicators of each headache category			
Initial diagnosis	Migraine or MOH^a^	TTH^a^	TACs^a^	Other primary headaches^a^	Other headaches^a^	Total	Accuracy rate (%)	Sensitivity (%)	Specificity (%)	Positive predictive value (%)
Unconfirmed^b^	9,208	2,753	33	90	15	12,099	-	-	-	-
Migraine or MOH^b^	84,021	724	42	27	3	84,817	90.5	89.0	96.6	99.1
TTH^b^	1,111	19,229	2	8	0	20,350	96.1	84.6	98.8	94.5
TACs^b^	43	7	235	1	0	286	99.9	75.3	99.96	82.2
Other primary headaches^b^	37	21	0	500	0	558	99.8	79.9	99.95	89.6
Other headaches^b^	5	1	0	0	41	47	99.98	69.5	99.99	87.2
Total	94,425	22,735	312	626	59	110,157	88.0	-	-	-

**Table 6:** Consistency of initial headache diagnoses and diagnoses after 7–12 months ^a^Subsequent diagnoses were defined as the latest diagnosis recorded between 7–12 months after the initial diagnosis. ^b^We classified headache diagnoses into five categories based on the ICD-10 codes: unconfirmed (R51, G44), migraine and medication-overuse headache (MOH); (G43, G430, G431, G432, G433, G438, G444, G439), tension-type headache (TTH; G442), trigeminal autonomic cephalalgias (TACs; G440), other primary headaches (G448), other headaches including secondary headaches (G441, G443). Initial diagnoses included suspected diagnoses; however, subsequent diagnoses excluded suspected diagnoses.

	Diagnosis after 7–12 months^a^						Indicators of each headache category			
Initial diagnosis	Migraine or MOH^a^	TTH^b ^	TACs^b^	Other primary headaches^b ^	Other headaches^b ^	Total	Accuracy rate (%)	Sensitivity (%)	Specificity (%)	Positive predictive value (%)
Unconfirmed^b ^	14,990	4,607	58	143	29	19,827	-	-	-	-
Migraine or MOH^b^	82,088	1,216	51	41	3	83,399	85.4	83.0	94.8	98.4
TTH^b^	1,706	18,197	4	27	1	19,935	93.9	75.6	98.3	91.3
TACs^b ^	59	12	228	1	0	300	99.9	66.9	99.9	76.0
Other primary headaches^b^	67	34	0	486	0	587	99.7	69.6	99.9	82.8
Other headaches^b ^	2	0	0	0	26	28	99.97	44.1	99.99	92.9
Total	98,912	24,066	341	698	59	124,076	81.4	-	-	-

**Table 7:** Consistency of initial headache diagnoses and diagnoses after 12–36 months ^a^Subsequent diagnoses were defined as the latest diagnosis recorded between 13–36 months after the initial diagnosis. ^b^We classified headache diagnoses into five categories based on the ICD-10 codes: unconfirmed (R51, G44), migraine and medication-overuse headache (MOH); (G43, G430, G431, G432, G433, G438, G444, G439), tension-type headache (TTH; G442), trigeminal autonomic cephalalgias (TACs; G440), other primary headaches (G448), other headaches including secondary headaches (G441, G443). Initial diagnoses included suspected diagnoses; however, subsequent diagnoses excluded suspected diagnoses.

	Diagnosis after 12–36 months^a^						Indicators of each headache category				
Initial diagnosis	Migraine or MOH^b^	TTH^b^	TACs^b^	Other primary headaches^b^	Other headaches^b^	Total	Accuracy rate (%)	Sensitivity (%)	Specificity (%)	Positive predictive value (%)
Unconfirmed^b^	31,912	10,992	147	351	61	43,463	-	-	-	-
Migraine or MOH^b^	81,504	2,583	140	86	17	84,330	74.3	69.6	91.3	96.6
TTH^b^	3,367	17,145	15	52	5	20,584	88.5	55.7	97.1	83.3
TACs^b^	126	23	215	4	0	368	99.7	41.4	99.9	58.4
Other primary headaches^b^	113	57	1	399	0	570	99.6	44.7	99.9	70.0
Other headaches^b ^	11	8	1	1	18	39	99.9	17.8	99.99	46.2
Total	117,033	30,808	519	893	101	149,354	66.5	-	-	-

Overall accuracy rates decreased over time, being 90.8% at visits after one to three months, 88.0% after four to six months, 81.4% after seven to 12 months, and 66.5% after 13-36 months. When analyzed based on the headache category, the diagnostics trends were consistent with those seen after 37-60 months, with the accuracy rate and specificity of the migraine and MOH category being the lowest, while the sensitivity and PPV were the highest. Among the patients who received a specific diagnosis at their initial visit, the most common diagnostic change during any of the subsequent visits was from TTH to migraine or MOH.

Subgroup analyses 

**Figure 1:**
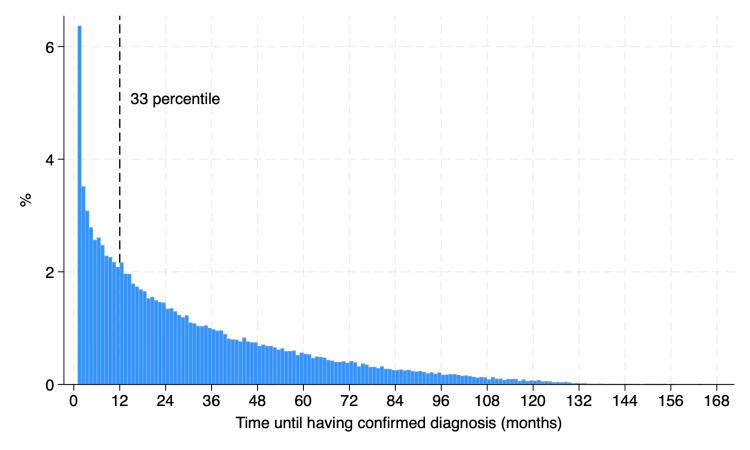
Time until a definitive headache diagnosis among patients whose initial diagnoses were unconfirmed. We calculated the time until a definitive diagnosis among patients who received an unconfirmed diagnosis after their initial visit. Over one in three patients (n = 32,974, 34.4%) received confirmed diagnosis within 12 months after their unconfirmed initial diagnosis.

Among the patients whose initial diagnosis was unconfirmed, approximately one in three (n=28,333, 29.6%) visited medical institutions the next month. The mean length of time until they received a definitive diagnosis was 30.4 months (SD 28.0), and one in three had a definitive diagnosis within 12 months after their initial visit (Figure 1).

As shown in Table 8, the most common definitive diagnosis for these patients was migraine and MOH (n = 68,071, 71.0%), followed by TTH (n=26,449, 27.6%), other primary headaches (n=863, 0.9%), TACs (n=339, 0.4%), and other headaches (n=168, 0.2%). 

**Table 8:** Definitive diagnoses among patients with unconfirmed diagnoses at their initial visit. ^a^We classified headache diagnoses into five categories based on the ICD-10 codes: unconfirmed (R51, G44), migraine and medication-overuse headache (MOH); (G43, G430, G431, G432, G433, G438, G444, G439), tension-type headache (TTH; G442), trigeminal autonomic cephalalgias (TACs; G440), other primary headaches (G448), other headaches including secondary headaches (G441, G443).

	Definitive diagnosis					
	Migraine or MOH^a ^	TTH^a^	TACs^a^	Other primary headaches^a^	Other headaches^a^	Total
No. of patients (%)	68,071 (71.0)	26,449 (27.6)	339 (0.4)	863 (0.9)	168 (0.2)	95,890
Time until having confirmed diagnosis, mean, (SD), months	29.7 (27.7)	32.0 (28.6)	30.3 (27.7)	32.8 (29.6)	34.9 (29.9)	30.4 (28.0)

## Discussion

This study found that more than one in four patients received an unconfirmed diagnosis at their initial visit, and that headache diagnoses often changed over time. Migraine or MOH was the most common diagnosis across all periods, followed by TTH. Three to five years after the initial visit, over 40% of patients received diagnoses that differed from those received at their initial visit. Diagnostic changes from TTH to migraine or MOH were the most common.

We could not assess which diagnoses were correct in cases in which different diagnoses were given at the initial and subsequent visits, as information on the diagnostic processes and patient symptoms were not available. Here we discuss some possible explanations for the decrease in the consistency of headache diagnoses over time.

Initial misdiagnosis due to limited information and non-specialist assessment

An initial diagnosis may be incorrect, and physicians may be more likely to make a correct diagnosis at a subsequent visit as they monitor and manage a patient’s headaches over time. Previous research indicates there is a high misdiagnosis rate for primary headaches during initial patient visits, with only 42% of patients with migraines receiving migraine diagnoses at their initial physician visit in Turkey [20]. The diagnostic challenges associated with these initial visits may be attributed to the scarce medical information available during these visits, with headache diagnoses being solely based on a physician’s clinical assessment due to a lack of diagnostic tests and biomarkers [7]. Headache diaries, which document headache patterns and frequency, and associated symptoms such as photophobia and nausea, are useful diagnostic tools [1]. Thus, physicians may find it easier to diagnose primary headaches correctly at subsequent visits when more comprehensive information from headache diaries and treatment responses are available.

Our study found that the most common diagnostic change at any point in time was from a TTH diagnosis to a migraine or MOH diagnosis. TTH and migraine are the two most prevalent types of primary headaches; however, differentiating between them can be challenging, particularly for non-specialists. Although the ICHD-3 differentiates them clearly, non-specialists may find it difficult to use it or not be familiar with using it. Additionally, migraines and TTHs have similar clinical clinical presentations in some cases, which can lead to misdiagnosis [21]. For example, migraines and TTHs can be provoked and exacerbated by similar psychological factors, such as stress, anxiety, and depressive symptoms. Nausea is common in patients with migraine, and some chronic TTH patients experience mild nausea [1,21].

A study in the United States found that 32% of patients who were initially diagnosed with TTH were later diagnosed with migraines after keeping headache diaries [22]. In the current study, we could not obtain the data regarding the physicians’ board certification; however, most of the diagnoses included in the study were likely to be made by non-specialists. This assumption is based on the small number of headache specialists in Japan, with 1,037 board-certified headache specialists as of 2025 March [23]. Therefore, based on the above assumptions, the correct diagnosis rate during initial visits made by non-specialists may be less than 60% in Japan. This indicates that it may take time to make an accurate diagnosis, particularly for non-specialists.

Coexisting or shifting headache types within the same patient

The discrepancy in diagnoses could be due to patients having other types of concomitant headaches over time. Some headache conditions, such as migraines and TTHs, can coexist, contributing to complexity associated with headache diagnoses [21]. In addition, one patient may exhibit individual headache attacks that vary in presentation, with some resembling TTHs and others showing more typical migraine features. These diagnostic challenges are further compounded by the fact that patients may not recognize or report key migraine-associated symptoms, such as nausea, photophobia, and phonophobia, or the degree to which the headaches disrupt their daily lives. This may be particularly relevant in Japan, where cultural factors, such as a strong work ethic and reluctance to take sick leave, may result in individuals’ underestimating the impact of their headaches on their functioning [24].

Chronification and symptom evolution masking migraine features

Making correct diagnoses at a later visit may be more difficult because in some cases headaches can evolve into chronic headaches or be complicated by medication overuse due to inadequate pain management, leading to complex headache presentations [25,26]. For instance, in some patients with migraines, the condition may become chronic, particularly during middle age. In such instances, the frequency of headaches may increase to almost daily; however, their individual intensity may decrease. Symptoms such as photophobia, phonophobia, and nausea may become less prominent, and the pain may resemble TTHs. However, migraine-specific features often persist, such as exacerbation during menstruation or the presence of unilateral, pulsating pain. Conversely, some patients who are initially diagnosed with TTH may later be diagnosed with migraines as their symptoms become more severe or disabling, or as characteristic migraine features, such as pulsating pain, nausea, or sensitivity to light and sound, emerge over time. These evolving symptoms can complicate the diagnostic processes over time, particularly in non-specialist settings.

Challenges associated with headache diagnosis in Japan and the need for system-level interventions

We found that most patients visited clinics without inpatient beds, and over one in four patients received an unconfirmed headache diagnosis during their initial visit. This indicates that many patients went to the primary care clinics rather than hospitals with headache specialists, highlighting the challenges faced by physicians in making timely and accurate headache diagnoses. While the frequency of undiagnosed headaches varies in other countries, it is common for patients to not receive a definitive diagnosis at their initial visit. Previous studies reported that 70% of patients with headaches did not receive a diagnosis in UK primary clinics [9], while over half of the patients with primary headaches (56.6%) remain undiagnosed in China [11]. This study is the first large-scale study to demonstrate that many headache patients are initially undiagnosed in Japan and highlights the need for interventions to help physicians diagnose headache in a timely and accurate manner.

Furthermore, our findings highlight the significant underdiagnosis of migraines. The most common diagnostic shift was from TTHs to migraines, indicating that many patients with migraines may be misdiagnosed initially. This is concerning as migraines are highly prevalent and are associated with considerable disability and socioeconomic burden, particularly among working-age adults. Delayed or inadequate treatment can lead to chronification and MOH, both of which are challenging and expensive to manage.

These challenges may be attributed to the limited opportunity to learn primary headache management for non-specialists. In Japan, many physicians are primarily trained to differentiate life-threatening or urgent causes of headaches, such as subarachnoid hemorrhage or brain tumors during their residency. As a result, they may receive limited training in differentiating among non-emergent primary headache disorders, such as migraines and TTHs. Meanwhile, the number of headache specialists remains insufficient to meet the population-level demand. Consequently, non-specialist physicians must play a greater role in the accurate diagnosis and management of primary headache disorders. To address these issues, system-level interventions are urgently needed. These could include providing training opportunities to non-specialists, implementing validated screening tools [27], integrating artificial intelligence-based diagnostic support in primary care [28], and promoting community-based awareness campaigns [29] to encourage the timely consultation and reporting of migraine-related symptoms.

Limitations 

This study has several limitations. First, although the ICDH-3 is commonly used to diagnose headaches, we used the ICD-10 codes, which might have resulted in the misclassifications of some headache diagnoses. Second, the recorded headache diagnosis in the insurance claims data may have differed from the physicians’ actual diagnosis. Although validation studies investigating headache diagnoses in claims data are limited, previous studies suggest that using diagnostic records of migraine or TTH has higher sensitivity, so it can be useful for estimating disease prevalence or incidence, whereas combining prescription and diagnostic records achieves a higher positive predictive value, which may be useful for estimating relative risk [30]. In this study, we used the diagnostic records alone, as the aim was to assess the changes in diagnoses over time across a broader population or patients with headaches.

Third, sampling bias may be present because the REZULT database contains employer-based insurance claims data. This could have led to certain characteristics being over- or under-represented, such as a higher proportion of younger individuals being present in the database compared to the general population. Additionally, we could not track patients whose insurance changed to the insurance that was not included in the REZULT database. Fourth, unmeasured confounding factors may be present. For example, we analyzed data from July 2010 through November 2024, but external events, such as the COVID-19 pandemic, may have impacted diagnoses during specific periods. 

Fifth, we could not verify the accuracy of the headache diagnoses, as information regarding physicians’ board certifications and the diagnostic processes used (e.g., imaging and physical examinations) was unavailable. This lack of contextual clinical process data limits our ability to validate headache diagnoses. Although we assumed that the definitive diagnoses given at subsequent visits were correct and calculated the accuracy rate based on these, it is possible that these diagnoses were also incorrect. Therefore, our estimates of diagnostic accuracy may either overestimate or underestimate. Further research is required to explore the underlying causes of the diagnostic difficulties associated with headache disorders and should incorporate detailed clinical data, including physicians’ board certification, diagnostic process, and patient-reported symptoms.

## Conclusions

In the current study, over one in four patients were undiagnosed during their initial visit, and headache diagnoses often changed over time. A substantial number of patients who were initially diagnosed with TTHs were later diagnosed with migraines, indicating a significant underdiagnosis of migraines. These results indicate a persistent delay and difficulty in achieving accurate headache diagnoses in Japan. Interventions are required to improve the accuracy and timeliness of headache diagnoses to ensure that patients receive adequate treatment. Potential interventions include enhancing primary care physician training on headache disorders, promoting the use of standardized diagnostic tools, and implementing clinical decision support systems such as artificial intelligence. Additionally, increasing public awareness about migraine symptoms and encouraging early consultation may also contribute to more timely and accurate diagnoses.
